# An evaluation of longitudinal changes in serum uric acid levels and associated risk of cardio-metabolic events and renal function decline in gout

**DOI:** 10.1371/journal.pone.0193622

**Published:** 2018-02-28

**Authors:** Rishi J. Desai, Jessica M. Franklin, Julia Spoendlin-Allen, Daniel H. Solomon, Goodarz Danaei, Seoyoung C. Kim

**Affiliations:** 1 Division of Pharmacoepidemiology and Pharmacoeconomics, Brigham and Women’s Hospital & Harvard Medical School, Boston, Massachusetts, United States of America; 2 Division of Rheumatology, Immunology and Allergy, Brigham and Women’s Hospital, Boston, Massachusetts, United States of America; 3 Department of Global Health and Population, Harvard School of Public Health, Boston, Massachusetts, United States of America; The University of Tokyo, JAPAN

## Abstract

**Objective:**

Gout patients have a high burden of co-morbid conditions including diabetes mellitus (DM), chronic kidney disease (CKD), and cardiovascular disease (CVD). We sought to evaluate the association between changes in serum uric acid (SUA) levels over time and the risk of incident DM, CVD, and renal function decline in gout patients.

**Methods:**

An observational cohort study was conducted among enrollees of private health insurance programs in the US between 2004 and 2015. Gout patients were included on the index date of a SUA measurement ≥6.8 mg/dl. The exposure of interest was cumulative change in SUA levels from baseline. Hazard ratios (HR) and 95% confidence intervals (CI) for incident DM, incident CVD, and renal function decline (≥30% reduction in glomerular filtration rate) were derived using marginal structural models with stabilized inverse probability weights accounting for baseline confounders (age, gender, co-morbidities, co-medications) and time-varying confounders (serum creatinine, blood urea nitrogen, glycated hemoglobin).

**Results:**

Among 26,341 patients with gout, the average age was 62, 75% were men, and the median baseline SUA was 8.6 mg/dl (interquartile range 7.7 to 9.5). The incidence rates/100 person-years (95% CI) were 1.63 (1.51–1.75) for DM, 0.77 (0.70–0.84) for CVD, and 4.32 (4.14–4.49) for renal function decline. The adjusted HR (95% CI) per 3 mg/dl reduction in SUA, corresponding on average to achieving the target level of <6 mg/dl in this population, was 1.04 (0.92–1.17) for DM, 1.07 (0.89–1.29) for CVD, and 0.85 (0.78–0.92) for renal function decline.

**Conclusions:**

Reduction in SUA in patients with gout may be associated with a reduced risk of renal function decline, but not with DM or CVD.

## Introduction

Gout is the most common inflammatory arthritis affecting approximately 8.3 million Americans, and the incidence is increasing over time.[1–4] Acute gout is triggered by uric acid crystallization within the joints and lowering serum uric acid to subsaturating levels (≤6.8 mg/dl) to prevent crystal deposition is the primary approach of managing gout.[5] Many epidemiologic studies report that gout and hyperuricemia are associated with an increased risk of diabetes mellitus (DM), chronic kidney disease (CKD), cardiovascular disease (CVD) including myocardial infarction (MI) and stroke, and mortality.[6–14] Insight into managing the increased risk for these events is urgently needed to improve outcomes in patients with gout.

Based on the consistently reported associations between high levels of serum uric acid and cardio-metabolic and renal events, it has been hypothesized that lowering the levels of serum uric acid in gout patients may reduce the excess risk of these events.[15, 16] However, the evidence directly testing this hypothesis is limited and conflicting. A majority of the evidence testing this hypothesis comes indirectly from studies suggesting a potential association between allopurinol, which is a urate lowering agent acting through inhibition of xanthine oxidase, and lowered risk of cardiovascular and renal events.[17–21] However, results from some other studies have observed no benefit of xanthine oxidase inhibitors on cardio-metabolic risks in gout.[22, 23] More recently, a large Mendelian randomization study observed no association between 28 single nucleotide polymorphisms known to regulate serum uric acid levels and DM, CVD, and heart failure, raising questions about the causal role of serum uric acid in development of these events.[24]

To add critical data to the ongoing debate of whether aggressively lowering serum uric acid in patient with gout can lower the risk of future cardio-metabolic and renal events, we designed a large observational cohort study using a comprehensive database containing information on patients’ laboratory test results combined with their health insurance claims. The primary objective of our study was to evaluate the association between cumulative changes in serum uric acid levels over time and the risk of DM, CVD, and worsening of renal function in patients with gout.

## Material and methods

### Study design and data source

An observational cohort study was conducted using de-identified data from Clinformatics ^™^ Datamart (OptumInsight, Eden Prairie, MN) database for the period of January, 2004 to September, 2015. We used the subset of this database where health insurance claims data are combined with laboratory test results for enrollees in a large US commercial insurance program. Comprehensive longitudinal information on medical diagnoses, procedures, hospitalizations, outpatient visits, and pharmacy dispensing in addition to laboratory test results are available in this data source. The study database was de-identified to protect patients’ privacy and the study protocol was approved by the Institutional Review Board of the Brigham and Women’s Hospital.

### Study population and eligibility criteria

The potentially eligible study population consisted of gout patients at least 40 years of age who were continuously enrolled in their health plan for ≥6 months before a recorded serum uric acid measurement of 6.8 mg/dl or higher. Date of this uric acid measurement was defined as the index date. We excluded patients with prevalent use of urate lowering treatments including xanthine oxidase inhibitors, probenecid, or pegloticase, in the 6 months pre-index period to minimize confounding by duration of these treatments and reliably model the trajectory of uric acid changes over time in the target population of gout patients eligible for initiation of urate lowering treatments. We further excluded patients with cancer, end-stage renal disease, or renal transplant any time prior to the baseline uric acid measurement as their disease progression may be unique and not generalizable to the average gout patient. We also required availability of at least one test result for uric acid following the index measurement in order to calculate cumulative change in uric acid over time. Further, we imposed outcome-specific exclusion criteria in this base study population where patients with pre-existing DM were excluded from the analysis of incident DM risk, patients with pre-existing CVD or heart failure[25, 26] were excluded from the analysis of incident CVD risk, and patients for whom at least one pre-index and one post-index serum creatinine results were not available to estimate renal function decline were excluded from the analysis of renal function decline.

### Measurement of cumulative changes in serum uric acid levels

Changes in serum uric acid levels were measured as time-varying variable updated monthly whenever new results were available and carried forward from the most recently recorded value when unavailable. The main exposure variable of interest was defined as cumulative change in serum uric acid levels at the beginning of every month as a sum of all changes up to that point from the index measurement. Please refer to S1 Fig. for a graphical representation of the study design and exposure measurement.

### Outcome measurement

Outcomes were measured beginning on day 31 post-index until patients’ disenrollment from their health plan or the last date of data availability (30^th^ September, 2015), whichever came first. We conducted analyses for three separate outcomes: 1) incident DM defined as HbA1c levels of ≥ 7 or a combination of a diagnosis code and filled prescription of a hypoglycemic agent, 2) incident CVD defined as an inpatient diagnosis of MI or ischemic stroke in the primary position or procedure codes indicating stenting or coronary artery bypass surgery during a hospitalization,[25, 27] and 3) worsening of renal function defined as reduction in the estimated glomerular filtration rate (eGFR) of ≥ 30% from the baseline measurement.[28] eGFR values were calculated using the Modification of Diet in Renal Disease (MDRD) equation from recorded serum creatinine results without correction for race as we did not have information on patient race in our database.[29]

### Covariates

We assessed a wide range of covariates at baseline including age, gender, known cardiovascular risk factors and co-medications (hyperlipidemia, hypertension, obesity, smoking, alcohol abuse, sleep apnea, chronic kidney disease, antihypertensive medications, lipid lowering agents, and antiplatelet agents) for all three analyses. We also included analysis-specific covariates (i.e., MI, stroke, heart failure, and previous revascularization procedures for the DM and renal function analyses; DM, hypoglycemic treatment use, and levels of HbA1c for incident CVD and renal function analyses).

Additionally, use of non-steroidal anti-inflammatory drugs (NSAIDs), steroids, colchicine, statins, laboratory test results [serum creatinine, blood urea nitrogen, and HbA1c (for incident CVD and renal function analysis only)], and healthcare use factors (number of distinct medications, office visits, emergency room visits, hospitalizations) were also considered time-varying covariates. All time-varying covariates were updated monthly.

### Sensitivity analyses

Use of urate lowering therapy (xanthine oxidase inhibitors, pegloticase, and probenecid) is the primary mechanism to lower serum uric acid levels; however, other mechanisms such as diet and genetic factors may also play a role. Adjusting for any of these mechanisms in the analysis potentially violates the consistency assumption of causal inference and results in estimates for isolated effects of an implied intervention to lower uric acid on the risk of cardiometabolic and renal events.[30] In other words, adjusting for urate lowering therapy in this analysis provides estimates for the association between serum uric acid lowering through treatment-independent mechanisms and risk of outcomes. Therefore, in the primary analysis, we did not adjust for urate lowering treatment use during follow-up, but in a sensitivity analysis, we provided estimates after adjusting for time-varying urate lowering treatments.

In another sensitivity analysis, we provided estimates for the association between changes in uric acid over time and renal function decline among patients who were not on Renin Angiotensin System inhibitors, since both Angiotensin converting enzyme (ACE)-inhibitors and Angiotensin receptor blockers (ARBs), are known to slow the progression of renal function decline through reduction of proteinuria.[31]

### Statistical analysis

To appropriately account for time-varying confounding in the association between cumulative change in serum uric acid and cardio-metabolic and renal events of interest, we used marginal structural models with stabilized inverse probability of exposure weights.[32, 33] These weights were calculated monthly during the follow-up as a ratio of two subject-specific conditional probability density functions estimated form generalized linear models with cumulative serum uric acid change as the dependent variable: the numerator (the stabilizing factor) was the probability density of each subject’s own cumulative serum uric acid change conditional on time-fixed covariates, while the denominator was the probability density of each subject’s own cumulative serum uric acid change conditional on time-fixed covariates and time-varying covariates.[34] We assumed both density functions to have normal distribution and constant variance. To prevent variance inflation due to outliers with extremely large weights, we truncated the weights at the 1^st^ and the 99^th^ percentiles.[35] Please refer to S1 Fig. for technical details of weight calculation.

After truncation of the weights, a weighted pooled logistic regression model was specified to derive estimates for the effect of cumulative change in serum uric acid and the risk in outcomes of interest. Parameters of this weighted pooled logistic model estimate hazard ratios (HR) of a marginal structural model.[32] The cumulative change variable was linearly modeled in the primary analysis. In a secondary analysis, restricted cubic spline modeling with 3 knots (at the 10^th^, 50^th^, and 90^th^ percentile of the distribution) was used to allow for non-linearity in the association between cumulative serum uric acid change and the risk of outcomes of interest. The weighted model adjusted for only time-fixed covariates since time-varying covariates were accounted for while constructing inverse probability weights. All HRs were reported along with 95% confidence intervals (CI) for 3 mg/dl reduction in serum uric acid levels to facilitate clinical interpretability of these estimates. Reduction of this magnitude would correspond to average serum uric acid levels of <6 mg/dl in our cohort of gout patients (average baseline serum uric acid levels 8.6 mg/dl), which is recommended by rheumatology societies as the target level in gout.[36, 37] To account for repeated measures over time in the same patients, we used a robust variance estimator to estimate CIs. All the analyses were conducted using SAS 9.3 (SAS Institute, Cary, NC).

## Results

### Cohort selection

**Fig 1:**
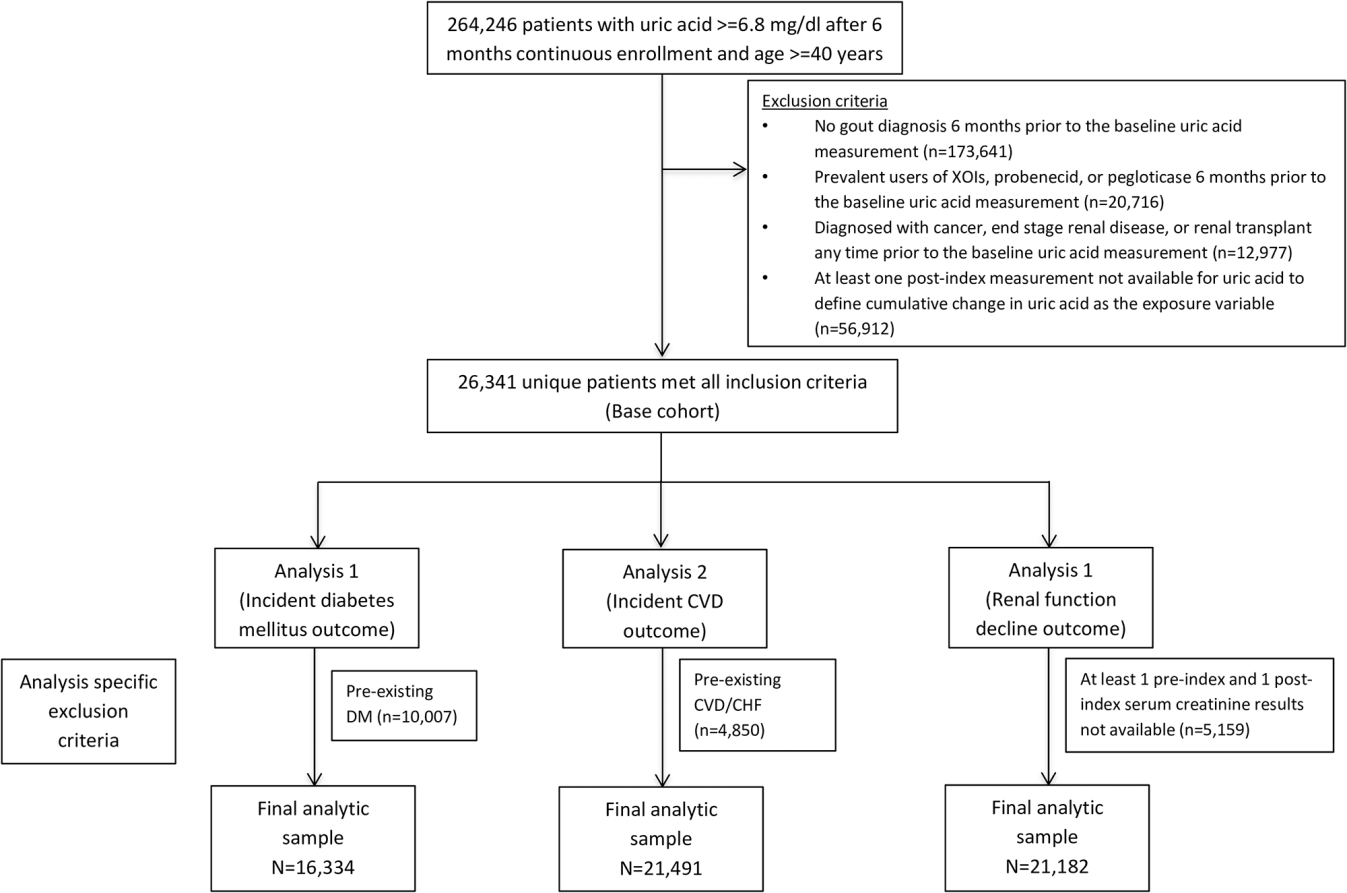
Cohort selection flow-chart, Clinformatics ^™^ Datamart 2004–2015. Abbreviations: CVD- Cardiovascular, DM- diabetes mellitus, XOI- Xanthine oxidase inhibitor.

We first identified 26,341 unique gout patients meeting all our inclusion criteria upon a recorded serum uric acid measurement of 6.8 mg/dl or higher. Of these patients, we included 16,334 non-diabetic patients for the analysis of incident DM risk; 21,491 patients with no recorded CVD, CHF, or revascularization procedure for the analysis of incident CVD risk; and 21,182 patients with recorded results for serum creatinine at least twice (once in the baseline and once in the follow-up period) for the analysis of renal function decline (Fig 1).

### Patient characteristics

A majority of the patients in all three sub-cohorts were male and the average age was approximately 60 years. Average serum uric acid levels at baseline were 8.6 mg/dl for the DM and CVD analysis sub-cohorts and 8.7 mg/dl for the renal functional decline sub-cohort. The prevalence of comorbidities including hypertension, hyperlipidemia, and chronic kidney disease was highest in the renal function decline sub-cohort due to inclusion of patients with prevalent DM and CVD unlike the other two sub-cohorts. Gout related medication use including NSAIDs, steroids and colchicine was common in all three sub-cohorts (Table 1).

**Table 1:** Baseline characteristics of gout patients included in the cohort, Clinformatics ^™^ Datamart 2004–2015.

	Cohort for incident diabetes mellitus analysis (n = 16,334)	Cohort for incident CVD analysis (n = 21,491)	Cohort for renal decline analysis (n = 21,182)
**Demographics**			
Gender	13425 (82.2)	16896 (78.6)	15813 (74.7)
Age (Mean ± SD, years)	59.8 ± 12.2	59.7 ± 11.7	62.7 ± 12.2
**Laboratory results**			
Uric acid (Mean ± SD, mg/dl)	8.6 ± 1.3	8.6 ± 1.3	8.7 ± 1.4
Serum creatinine (Mean ± SD, mg/dl)	1.2 ± 0.6	1.2 ± 0.3	1.2 ± 0.4
Blood urea nitrogen (Mean ± SD, mg/dl)	18.1 ± 6.5	19.5 ± 7.8	20.9 ± 9.7
HbA1c among those with diabetes			
<6.5	0 (0)	2173 (10.1)	2734 (12.9)
6.5–9	0 (0)	2376 (11.1)	3086 (14.6)
> = 9	0 (0)	230 (1.1)	323 (1.5)
Not recorded	0 (0)	2269 (10.6)	2423 (11.4)
**Co-morbid diagnoses**			
Myocardial infarction	297 (1.8)	0 (0)	623 (2.9)
Stroke or transient ischemic events	711 (4.4)	0 (0)	1404 (6.6)
Revascularization procedures	315 (1.9)	0 (0)	640 (3)
Diabetes mellitus	0 (0)	7048 (32.9)	8566 (40.4)
Angina	950 (5.8)	964 (4.5)	1938 (9.1)
Hypertension	12002 (73.5)	16695 (77.7)	17704 (83.6)
Hyperlipidemia	11989 (73.4)	16763 (78)	17495 (82.6)
Obesity	2579 (15.8)	4091 (19)	4635 (21.9)
Smoking	1560 (9.6)	1806 (8.4)	2059 (9.7)
Alcohol abuse	155 (0.9)	169 (0.8)	216 (1)
Sleep apnea	1187 (7.3)	1718 (8)	2139 (10.1)
Chronic kidney disease	2660 (16.3)	4051 (18.8)	5838 (27.6)
**Medication use**			
ACE-inhibitors	4173 (25.5)	6019 (28)	6586 (31.1)
Angiotensin receptor blockers	2481 (15.2)	3784 (17.6)	4076 (19.2)
Anticoagulants	720 (4.4)	667 (3.1)	1248 (5.9)
Antiplatelets	502 (3.1)	438 (2)	1121 (5.3)
Beta-blockers	3396 (20.8)	4583 (21.3)	5375 (25.4)
Calcium channel blockers	3186 (19.5)	4592 (21.4)	5188 (24.5)
Diuretics	5288 (32.4)	7501 (34.9)	8698 (41.1)
Statins	4722 (28.9)	7099 (33)	8048 (38)
Other lipid lowering agents	1215 (7.4)	1857 (8.6)	2055 (9.7)
Oral hypoglycemics	0 (0)	3711 (17.3)	4582 (21.6)
Insulin	0 (0)	734 (3.4)	1234 (5.8)
Non-selective NSAIDs	6589 (40.3)	8531 (39.7)	7460 (35.2)
Cox-II inhibitors	386 (2.4)	514 (2.4)	510 (2.4)
Oral steroids	3827 (23.4)	4682 (21.8)	4678 (22.1)
Colchicine	3878 (23.7)	4969 (23.1)	4986 (23.5)

Abbreviations: ACE- angiotensin converting enzyme, CVD- Cardiovascular disease, NSAID- non-steroidal anti-inflammatory drug, SD- standard deviation.

### Cumulative changes in serum uric acid during follow-up

The average follow-up in the DM and CVD sub-cohorts was 2.8 years, while it was 2.6 years in the renal function decline sub-cohort. The median (interquartile range) cumulative reduction in serum uric acid during follow-up were 1 mg/dl (0 mg/dl, 2.6 mg/dl) in the DM analysis sub-cohort, 1 mg/dl (0 mg/dl, 2.7 mg/dl) in the CVD analysis sub-cohort, and 0.9 mg/dl (0 mg/dl, 2.6 mg/dl) in the renal functional decline analysis sub-cohort.

Over the follow-up period, 61% of the patients in the DM sub-cohort, 60% in the CVD sub-cohort, and 57% in the renal function decline sub-cohort were treated with a urate lowering agent. The mean cumulative exposure (± SD) to urate lowering treatment over the follow-up period was 10 (±15) months in the DM sub-cohort, 10 (±15) months in the CVD sub-cohort, and 9 (±14) months in the renal function decline sub-cohort. In line with expectations, the average cumulative reductions in serum uric acid levels were highly correlated with the duration of cumulative urate lowering treatment during follow-up (S2 Fig).

### Incidence rates for the outcomes

A total of 749 DM events, 470 CVD events, and 2,373 renal function decline events were observed with incidence rates/100 person-years (95% confidence intervals (CI)) of 1.63 (1.51–1.75), 0.77 (0.70–0.84), and 4.32 (4.14–4.49), respectively (Table 2).

**Table 2:** Incidence rates for the outcome events of interest in gout patients, Clinformatics ^™^ Datamart 2004–2015.

Outcome	Total sample size	Total person years of follow-up	Average follow-up years (SD)	Number of events	Incidence rates/100 person years (95% CI)
Diabetes mellitus	16,334	45,972	2.8 (2.0)	749	1.63 (1.51–1.75)
Composite cardiovascular endpoint (Myocardial infarction, Ischemic stroke, coronary revascularization)	21,491	60,910	2.8 (2.0)	470	0.77 (0.70–0.84)
Decline in renal function (30% reduction in estimated glomerular filtration rate from baseline)	21,182	54,981	2.6 (2.0)	2,373	4.32 (4.14–4.49)

### Association between cumulative serum uric acid change and outcomes

The overall mean (SD) MSM weights after truncation were for 1.00 (0.15) DM analysis, for 1.00 (0.22) CVD analysis, and for 1.00 (0.25) renal function decline analysis. Average weights at each follow-up time were also close to 1 and are presented for all three analyses in S3 Fig. In the primary analysis (Table 3), where cumulative change was modeled linearly, no association was observed between change in serum uric acid levels and the risk of DM (HR per 3 mg/dl reduction: 1.04, 95% CI 0.92–1.17) or incident CVD (HR per 3 mg/dl reduction: 1.09, 95% CI 0.89–1.29). However, reduction in serum uric acid was associated with a lower risk of renal function decline (HR per 3 mg/dl reduction: 0.85, 95% CI 0.78–0.92).

**Table 3:** Association between cumulative changes in serum uric acid over time and outcome events of interest in gout patients, Clinformatics ^™^ Datamart 2004–2015.

Outcome	Hazard ratio (95% confidence interval) for 3 mg/dl reduction in serum uric acid during the follow-up period	
	Unadjusted estimates	Marginal structural model estimates
Diabetes mellitus	0.85 (0.76–0.94)	1.04 (0.92–1.17)
Composite cardiovascular endpoint (Myocardial infarction, Ischemic stroke, revascularization)	0.90 (0.79–1.03)	1.07 (0.89–1.29)
Decline in renal function (30% reduction in glomerular filtration rate from baseline)	0.68 (0.63–0.72)	0.85 (0.78–0.92)

The estimates were consistent in a sensitivity analysis adjusting for time-varying use of urate lowering treatment (adjusted HR (95% CI) per 3 mg/dl reduction in SUA: 1.07 (0.91–1.24) for DM, 1.01 (0.81–1.27) for CVD, and 0.89 (0.81–0.98) for renal function decline). In the sensitivity analysis conducted among patients not on ACE-inhibitors or ARBs at baseline, the association between 3 mg/dl reduction in SUA and renal function decline was consistent with the primary analysis (adjusted HR, 95% CI: 0.82, 0.72–0.94).

**Fig 2:**
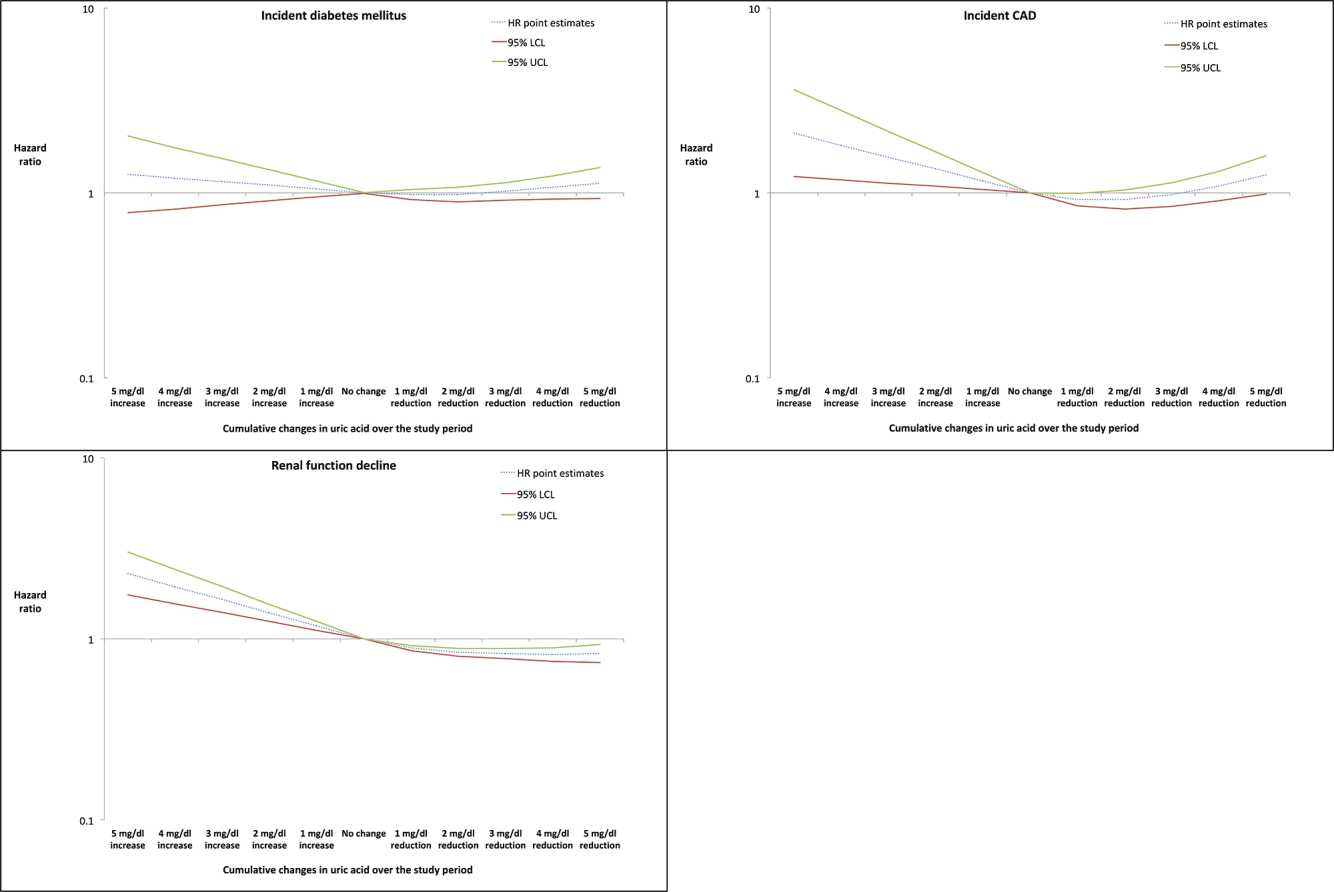
Association bewteen cumulative serum uric acid changes modeled as restricted cubic splines and cardiometabolic and renal outcomes in gout patients. Abbreviations: CVD- Cardiovascular disease, LCL- lower confidence limit, UCL- upper confidence limit.

Results of the secondary analysis, where cumulative changes in serum uric acid during follow-up were modeled as restricted cubic splines, are presented in Fig 2. Reductions in serum uric acid during follow-up were not associated with a lower risk of DM (HR for reduction by 3 mg/dl: 1.02, 95% CI 0.91–1.14). For incident CVD, increases in serum uric acid during follow-up were associated with a significantly increased risk (HR for increase by 3 mg/dl: 1.56, 95% CI 1.13–2.16). However, reductions in serum uric acid were not consistently associated with a reduced risk (HR for reduction by 3 mg/dl: 0.98, 95% CI 0.85–1.14). For renal function decline, increases in serum uric acid during follow-up were consistently associated with a significant increase in the risk (HR for increase by 3 mg/dl: 1.65, 95% CI 1.40–1.95) and reductions were associated with a significant decrease in risk (HR for reduction by 3 mg/dl: 0.83, 95% CI 0.78–0.89).

## Discussion

In this large observational study of 26,341 gout patients with serum uric acid measurement of 6.8 mg/dl or higher, we noted only a modest cumulative reduction in serum uric acid of about 1 mg/dl on average over the follow-up period of more than 2 years. Further, we did not find an association between cumulative reduction in serum uric acid in patients with gout and reduced risk of DM or incident CVD. However, reductions in serum uric acid were associated with a lower risk of renal function decline.

Multiple previous investigations have reported a positive association between serum uric acid levels and the risk of cardio-metabolic events. In a meta-analysis of 11 cohort studies, Kodama et al.[38] reported a 17% increased risk of DM with each unit increase in serum uric acid (RR 1.17, 95% CI 1.09–1.25). Similarly, in another meta-analysis of 26 studies, Kim et al.[9] reported a relative risk 1.12 (95% CI: 1.05–1.19) for cardiovascular mortality per unit increase in serum uric acid. Based on these observations, it has been hypothesized that lowering serum uric acid may have a role in prevention of DM and CVD.[39] However, using Mendelian randomization, a recent study demonstrated no association between genetically raised serum uric acid levels and DM (OR 0.95, 9%% CI 0.86–1.05), CVD (OR 1.02, 9%% CI 0.92–1.12), stroke (OR 0.99, 9%% CI 0.88–1.12), or heart failure (OR 1.07, 9%% CI 0.88–1.30).[24] The results from our current investigation suggesting no association between cumulative reductions in serum uric acid levels and DM as well CVD are completely in line with these findings. Our results, combined with findings from Keenan et al.,[24] suggest that serum uric acid may not be a causal factor in developing cardiometabolic events and may only be a marker for underlying risk factors for these events including, hyperinsulinemia, reduced renal function, and inflammation. It must also be noted that in the present investigation, we only included gout patients and the mean serum uric acid level at baseline was 8.6 mg/dl; which is by far greater than in the populations included in previous investigations.[9, 38] Therefore, it is possible that these patients may already have sustained substantial inflammatory burden and oxidative stress, which may not be completely and immediately reversible by simply lowering serum uric acid levels.

The association noted between reduction in serum uric acid levels and a reduced risk of renal function decline has potential clinical implications. According to a recent meta-analysis, 24% of the gout patients eventually develop CKD stage 3 or higher.[6] As CKD is strongly associated with end stage renal disease and all-cause mortality,[29] it is a major burden on the quality of life of the patients and imparts a substantial economic toll on the healthcare system. Our results suggest that patients with gout, particularly those who are identified as being at a greater risk for renal function decline, can benefit from aggressive management of serum uric acid with drug treatment and routine laboratory monitoring.

Our study also has some notable limitations. Most importantly, residual confounding is possible as we did not have information on some potential confounders including patient race, weight, blood pressure, and smoking intensity. Next, as with any observational study conducted using routine care data, we only were able to access laboratory results when the tests were ordered and recorded; this may result in exposure misclassification. Finally, our database included a relatively homogenous population of commercially insured patients and therefore, future studies should be considered to replicate our results in diverse populations including the elderly and patients with lower socioeconomic status. Future research focusing on evaluating whether different types of urate lowering treatments, namely Xanthine oxidase inhibitors, pegloticase, and probenecid, confer differential benefits in terms of renal function decline could be valuable to inform treatment decisions.

## Conclusions

In conclusion, in this large observational study, we observed that reduction in serum uric acid over time was associated with a reduced risk of renal function decline, but not DM or CVD, in hyperuricemic patients with gout. Our findings suggest that aggressive serum uric acid lowering approaches may slow renal disease progression but active management of traditional risk factors such as hypertension, hyperlipidemia, and obesity, as well as reducing the overall inflammatory burden may be required to manage the increased DM and CVD risk in gout patients.

## Supporting information

S1 Fig Study design summary and weight calculation for the marginal structural model. (PDF)

S2 Fig Average reductions in serum uric acid levels by months of cumulative exposure to urate lowering treatments. (PDF)

S3 Fig Weight distribution over time in each of the three study sub-cohorts. (PDF)
