# Supplementary material for: An evaluation of longitudinal changes in serum uric acid levels and associated risk of cardio-metabolic events and renal function decline in gout
Source: PLoS One. 2018 Feb 28;13(2):e0193622. doi: 10.1371/journal.pone.0193622 (PMC5831471; doi:10.1371/journal.pone.0193622)
Supplement: S1 Fig — (PDF) [file pone.0193622.s001.pdf]

eFigure 1: Study design summary and weight calculation for the marginal structural model

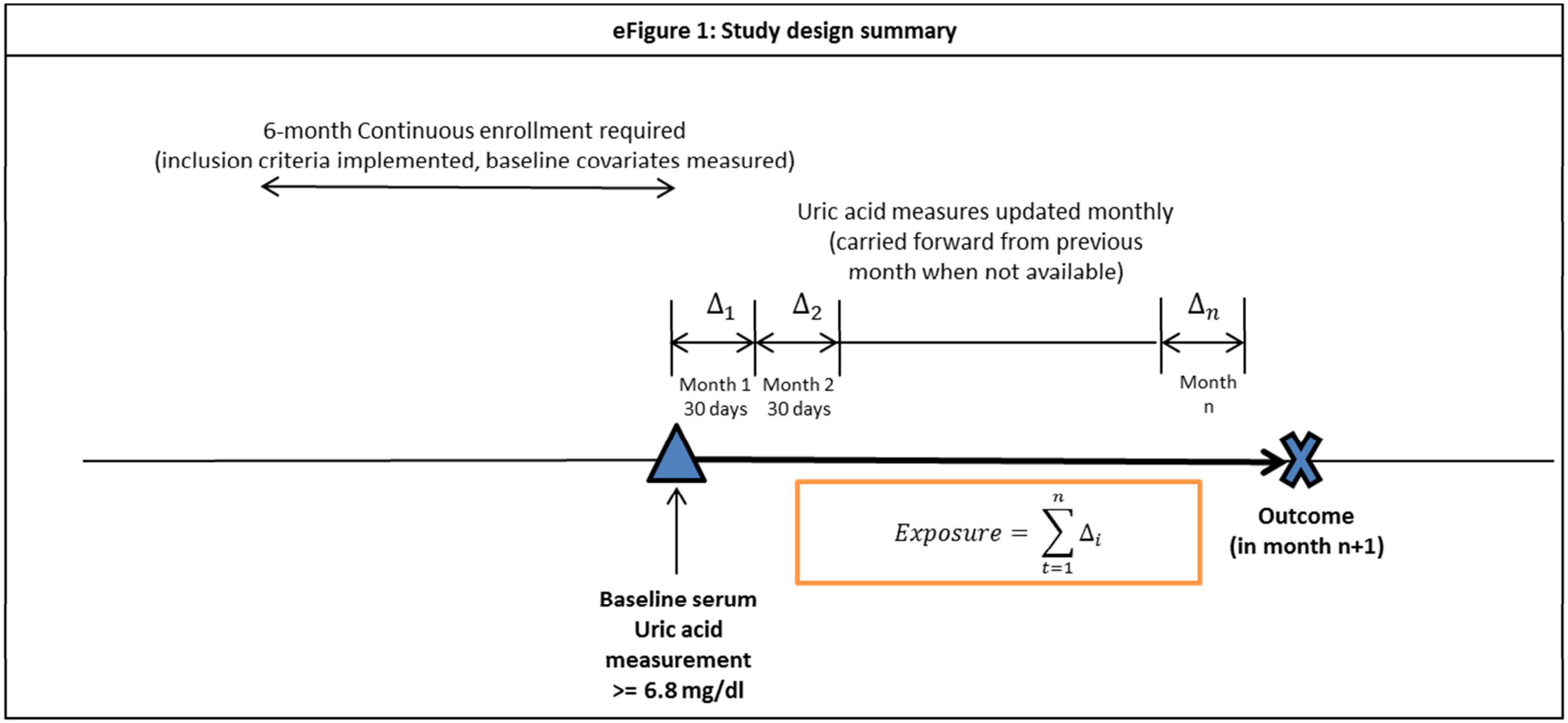

$$SW^{A}_{m+t} = \prod_{k=m}^{m+t} \frac{f(\Delta_k | \overline{\Delta}_{k-1}, L_0, \overline{D}_{k-1} = 0)}{f(\Delta_k | \overline{\Delta}_{k-1}, \overline{L}_k, \overline{D}_{k-1} = 0)}$$

Both the numerator and denominator are probability density functions calculated from generalized linear regression models (assumed normal distribution and constant variance)

Where:
m = Baseline month
t = Month of follow-up
Δ_k = cumulative change in serum uric acid in month k
$\overline{\Delta}_{k-1}$ = cumulative change in serum uric acid up to month k-1
L_0 = Covariates measured at baseline (ie Time-fixed)
$\overline{L}_k$ = Covariates measured at baseline (ie Time-fixed) + updated monthly (ie Time-varying)
($\overline{D}_{k-1}$ = 0) = Outcome not observed until month (k-1) and the patient is still at risk
