# Supplementary material for: An evaluation of longitudinal changes in serum uric acid levels and associated risk of cardio-metabolic events and renal function decline in gout
Source: PLoS One. 2018 Feb 28;13(2):e0193622. doi: 10.1371/journal.pone.0193622 (PMC5831471; doi:10.1371/journal.pone.0193622)

eFigure 2: Average reductions in serum uric acid levels by months of cumulative exposure to urate lowering treatments

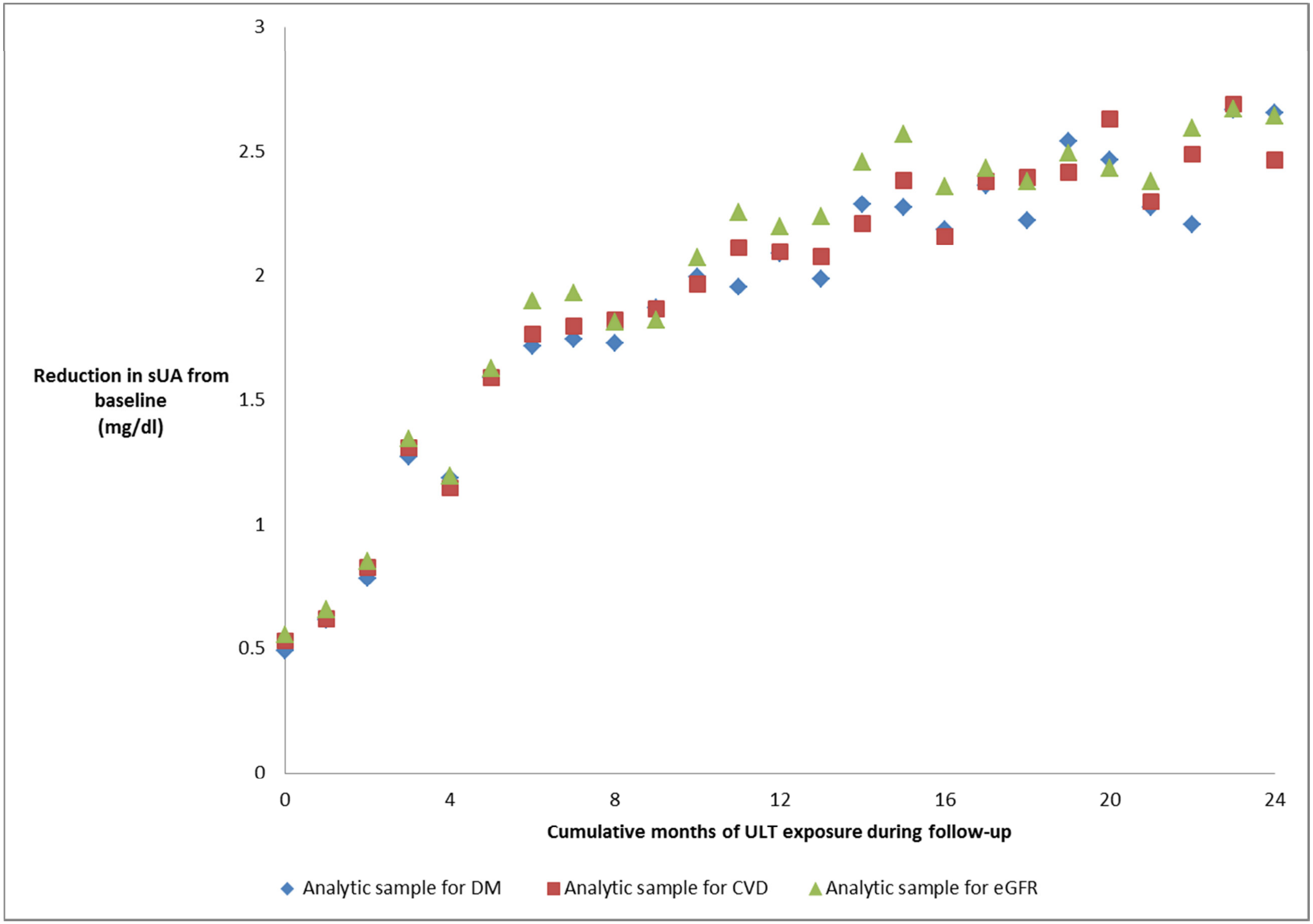

Supplement: S2 Fig — (PDF) [file pone.0193622.s002.pdf]
