# Supplementary figures and images for: An evaluation of longitudinal changes in serum uric acid levels and associated risk of cardio-metabolic events and renal function decline in gout
Source: PLoS One. 2018 Feb 28;13(2):e0193622. doi: 10.1371/journal.pone.0193622 (PMC5831471; doi:10.1371/journal.pone.0193622)

**eFigure 3: Weight distribution over time in each of the three study sub-cohorts**

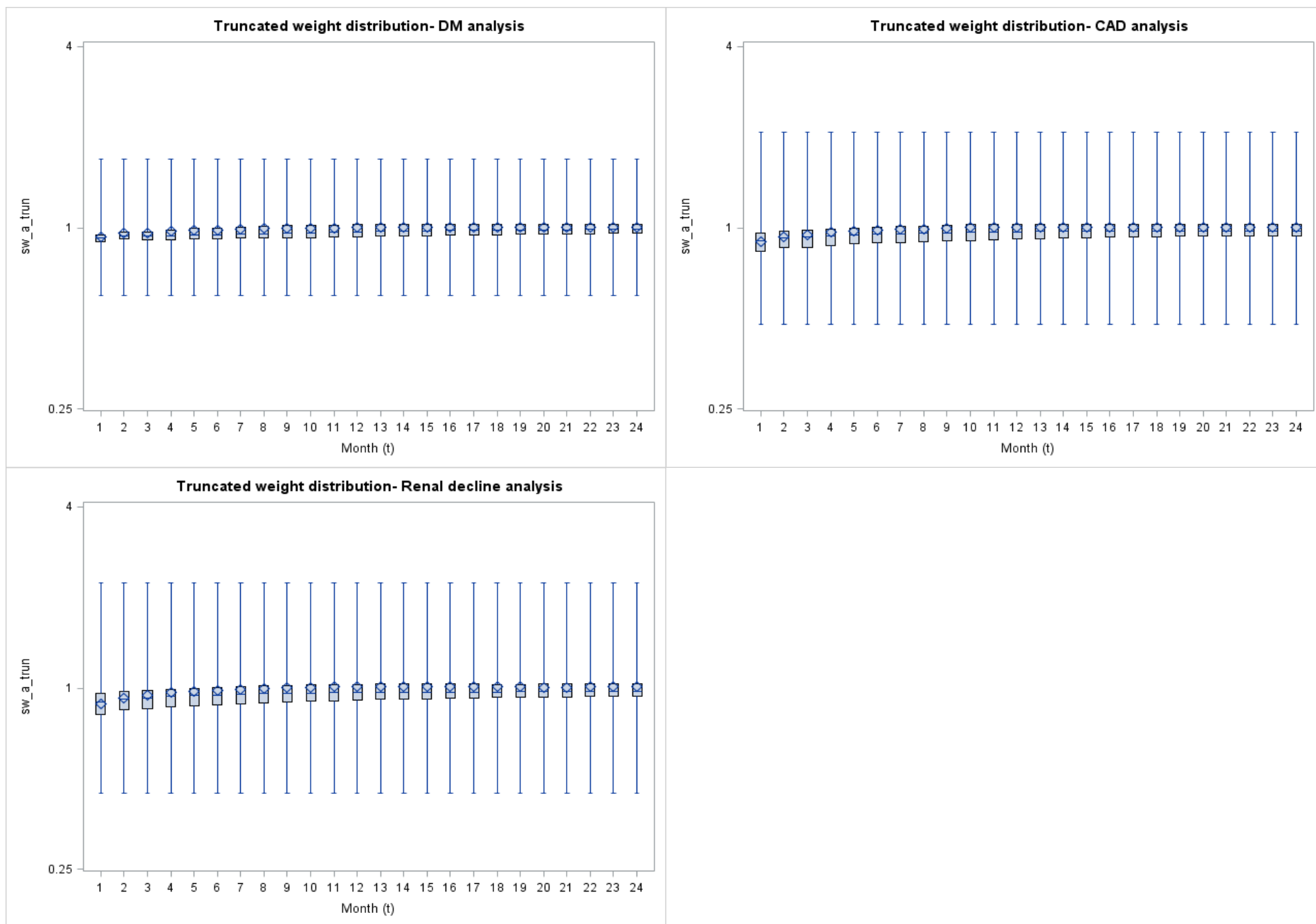

Supplement: S3 Fig — (PDF) [file pone.0193622.s003.pdf]
